# Fungal-mediated consolidated bioprocessing: the potential of *Fusarium oxysporum* for the lignocellulosic ethanol industry

**DOI:** 10.1186/s13568-016-0185-0

**Published:** 2016-02-18

**Authors:** Shahin S. Ali, Brian Nugent, Ewen Mullins, Fiona M. Doohan

**Affiliations:** 1 Molecular Plant–Microbe Interactions Laboratory, School of Biology and Environmental Science, University College Dublin, Dublin 4, Ireland; 2 SPCL, USDA/ARS Beltsville Agricultural Research Center, Beltsville, MD 20705 USA; 3 Department of Crop Science, Teagasc Research Centre, Oak Park, Carlow, Ireland

**Keywords:** Bioethanol, Fungi, Fusarium, Consolidated bioprocessing, Lignocellulose

## Abstract

Microbial bioprocessing of lignocellulose to bioethanol still poses challenges in terms of substrate catabolism. The most important challenge is to overcome substrate recalcitrance and to thus reduce the number of steps needed to biorefine lignocellulose. Conventionally, conversion involves chemical pretreatment of lignocellulose, followed by hydrolysis of biomass to monomer sugars that are subsequently fermented into bioethanol. Consolidated bioprocessing (CBP) has been suggested as an efficient and economical method of manufacturing bioethanol from lignocellulose. CBP integrates the hydrolysis and fermentation steps into a single process, thereby significantly reducing the amount of steps in the biorefining process. Filamentous fungi are remarkable organisms that are naturally specialised in deconstructing plant biomass and thus they have tremendous potential as components of CBP. The fungus *Fusarium oxysporum* has potential for CBP of lignocellulose to bioethanol. Here we discuss the complexity and potential of CBP, the bottlenecks in the process, and the potential influence of fungal genetic diversity, substrate complexity and new technologies on the efficacy of CPB of lignocellulose, with a focus on *F. oxysporum*.

## Background

Energy is the single most important commodity in the world today. In many ways, a nation’s success is largely dependent on their level of energy security. Obtaining a secure, renewable, environmentally-benign and cheap supply of energy is of crucial global importance. The world is highly dependent on fossil fuels for energy. The transportation sector in particular is almost entirely reliant on petroleum-based fuels. Energy use is also inexorably linked to climate change. In OECD countries, the transportation sector is responsible for 23 % of worldwide carbon dioxide emissions and over 70 % of global carbon monoxide emissions (International Energy Agency 2014). The substantial release of these greenhouse gases into the atmosphere has drastically quickened the rate of global warming and subsequent climate change. This rate of pollution shows no sign of abating and expectations are that there will be over 2 billion vehicles worldwide by 2030 (International Energy Agency 2015). This growth will put enormous stress on global ecosystems and the global climate (Balat 2011). The increasing demand and increases in atmospheric CO_2_ concentrations means that the continued use of fossil fuels at the current levels is clearly unsustainable. Therefore, the only way to stem this energy crisis is to reduce fossil fuel dependence and move to alternative fuels. With these factors in mind, the need for a cheaper, ‘greener’ and more self-sufficient energy source is of global importance.

For the past few decades, industry and governments have diversified research in an attempt to discover, develop and commercialise new technologies for alternative transportation fuels, including biofuels derived from plant biomass. Generous subsidies were given in many developed nations in order to stimulate the production of biofuel crops. But there are huge concerns regarding the increasing diversion of starch- or sucrose-rich crop materials and land from food to biofuel production. This has shifted attention to the use of lignocellulose-derived bioethanol as a biofuel (Morales et al. 2015; Himmel and Bayer 2009; Bentsen et al. 2014; Limayem and Ricke 2012; Banerjee et al. 2010). Lignocellulose is the structural backbone of plant material, mainly composed of cellulose and hemicelluloses linked by lignin. It is one of the most abundant materials on this planet but is generally not used as a human foodstuff.

**Fig. 1:**
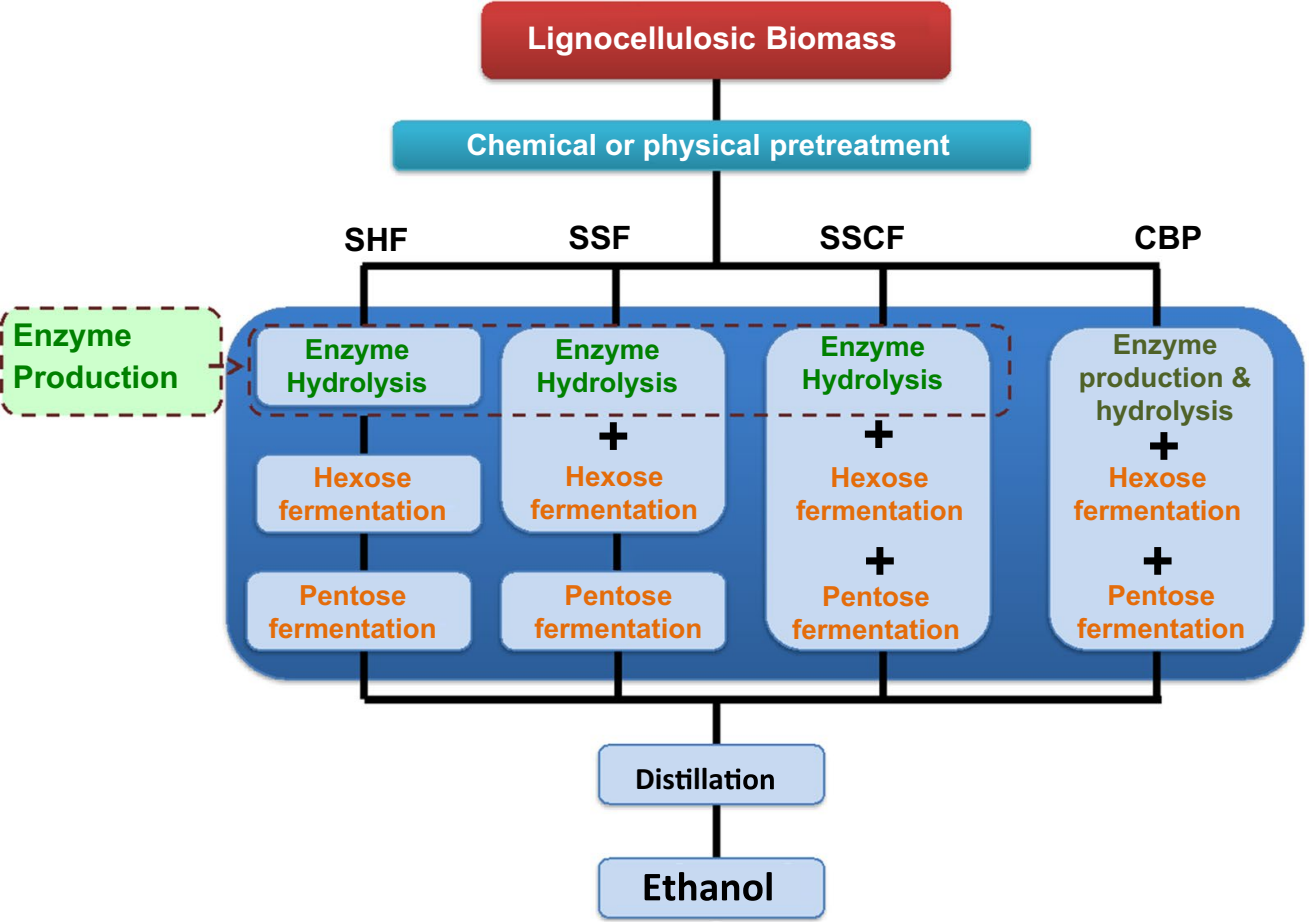
The steps involved in the bioprocessing to lignocellulose to ethanol. Following chemical pretreatment to break down the tough, recalcitrant material in lignocellulosic biomass, it is more susceptible to enzymatic attack because of the exposure of the underlying carbohydrates (i.e., cellulose and hemicelluloses). Thereafter, there are four possible routes to ethanol production; *SHF* separate hydrolysis and fermentation, *SSF* simultaneous saccharification and fermentation, *SSCF* simultaneous saccharification and co-fermentation, *CBP* consolidated bioprocessing. Microbial enzymes produced by bacteria, fungi and other micro-organisms are used to convert the exposed cellulose and hemicellulose sugar polymers to simple sugars which can then be efficiently fermented; in all cases except CBP, these enzymes need to be added (the CBP organism(s) both saccharify and ferment the substrate). Microbial fermentation is the final phase in the bioconversion process. By this stage, the hydrolysate contains a mixture of hexose and pentose sugars such as glucose, xylose, mannose, fructose, galactose and arabinose, which are all fermentable by micro-organisms to produce bioethanol

Despite having such advantages, lignocellulosic bioethanol cannot compete price-wise with starch or sucrose -based bioethanol (Balan et al. 2013; Guo et al. 2015). It’s tough molecular design has evolved over 400 million years and keeps plants upright and protects them from other organisms (Sanderson 2011). Converting lignocellulose into biofuel is costly; it requires several pretreatments, the enzymatic hydrolysis to release the sugars and the fermentation of sugars to bioethanol (Fig. 1). Several strategies can be used to convert lignocellulose to bioethanol. These include pretreatment followed by simultaneous saccharification and fermentation, simultaneous saccharification and co-fermentation and consolidated bioprocessing (CBP) (Li et al. 2014; Brethauer and Studer 2014; Lynd et al. 2008; Mosier et al. 2005; Xu et al. 2009). But, these processes are not yet commercially adopted. The current practice involves separate hydrolysis and fermentation, distinct steps in the process including the production of enzymes, biomass hydrolysis and subsequent fermentation of hexose and pentose sugars. However, the accumulation of high glucose content can inhibit some enzymes, principally glucosidases. Simultaneous saccharification and fermentation would circumvent this problem by preventing a build-up of glucose in the reactor (Olofsson et al. 2008). CBP is the ideal process, wherein the same microorganism is able to produce the enzymes, hydrolyse biomass and convert sugars into ethanol (Xu et al. 2009).

## Lignocellulosic bioethanol and current bottlenecks

Lignocellulosic material is the most abundant source of biomass on earth and includes wood, grasses, agricultural residues or any non-food-plant sources. The fermentable sugars are derived via the hydrolysis of the cellulose and hemicellulose components, which account for approximately 60 % of lignocellulosic materials, while lignin accounts for 15–25 % (Dionisi et al. 2015; Menon and Rao 2012; Wyman 1994). Lignocellulose offers several benefits over sugar and starch as a substrate for bioethanol production (Morales et al. 2015; Solomon et al. 2007). Most importantly, when lignocellulose is derived from non-food parts of plants, and as long as land use is not diverted to lignocellulose production, it does not interfere with food security (if the land use pattern is not changed).

Low carbon biofuels from commercial-scale cellulosic ethanol has become a reality in recent times. Numerous cellulosic ethanol refineries have now come online worldwide with several more in the pipeline (European Biofuels Technology Platform 2015). To date, the largest cellulosic ethanol industrial-scale refinery is the Beta Renewables/Novozyme funded plant situated at Crescentino in north-western Italy which commenced operations in October 2013. The facility is entirely self-sufficient, using the lignin and biogas by-products to power the plant which generates 75 million litres annually of cellulosic ethanol, enough fuel for more than 50,000 cars. A sister plant in Strazke, Slovak Republic is currently under construction while in Fuyang, China a biorefinery four times the capacity of the Crescentino plant is under development. In the US, there are over 200 corn-based ethanol plants in operation (Gnansounou 2010). Many of these bioethanol plants are evolving to become cellulosic ethanol production facilities utilising cheaper agricultural residues and non-food substrates. In October 2014 the Spanish renewable energy giant, Abengoa officially opened its 25 million gallons per year ethanol commercial biorefinery at Hugoton, Kansas. A similar-scaled plant financed through a collaborative venture between chemical group Du Pont and Murex LLC is also nearing completion in Nevada, Iowa. Encouraging yields ranging from 68 to 83 gallons per tonne of biomass have recently been reported by several bioenergy groups such as Abengoa Bioenergy, Iogen Energy and Poet, LLC from their respective pilot cellulosic ethanol plants (Guo et al. 2015). With the inevitable upsurge in oil price back to pre-2014 levels an unavoidable reality allied with advancements in the relevant technology, industrial-scale lignocellulosic bioethanol will continue to spread worldwide in the near future.

The nature of lignocellulose leads to several bottlenecks in improving the commercial viability of second generation bioethanol. These include the substrate recalcitrance to digestion, the lack of fermentability of some of the sugars released via hydrolysis, the substrate heterogeneity and the transportation costs. The transportation cost can be reduced by building bioethanol refineries close to biomass production sites (Eggeman and Elander 2005). The heterogeneity in both the chemistry and structure of lignocellulose means that it is often difficult to optimise the production process in terms of pretreatment, enzymatic hydrolysis and fermentation to suit the various types of lignocellulosic biomass. The pretreatment of lignocellulosic biomass to reveal the cellulose and hemicellulose is very expensive and contributes up to 33 % of the overall costs of producing lignocellulosic bioethanol (Behera et al. 2014; Tomás-Pejó et al. 2009). Hence the focus on the genetic manipulation of the lignin biosynthetic pathway in plants in order to improve bioethanol yields from lignocellulosic materials (Fu et al. 2011; Hopkins et al. 2007). Traditional breeding can also be used in order to breed plant genotypes with altered composition/degradability, as demonstrated for wheat (Ali et al. 2012a). The micro-organisms used in the fermentation process to date are incompetent at efficiently co-fermenting the variety of sugars released from lignocellulosic materials. Many microbial strains used in bioethanol production, including *Saccharomyces cerevisiae,* can successfully ferment glucose and other hexose sugars but struggle to adequately ferment pentose sugars such as xylose and arabinose (Gírio et al. 2010; Hahn-hägerdal et al. 2007; Hector et al. 2008). Xylose is the second most common sugar found in plants and the inability of current microbial strains to successfully ferment xylose is an immensely limiting factor. While several yeast strains have been genetically modified for improved xylose fermentation (Zha et al. 2014; Lee et al. 2012; Chu and Lee 2007; Eliasson et al. 2000; Ho et al. 1998; Kuyper et al. 2005; Laluce et al. 2012), a naturally-occurring yeast or fungal strain equipped with an independent xylose transporter has yet to be found (Hector et al. 2008). In the race towards making the lignocellulosic bioethanol economically viable, some researchers are using directed mutagenesis to improve the activity of enzymes in microbes currently used for bioconversion; others are trying to build the ultimate microbe in the laboratory that exhibits high efficiency in the processes of both saccharification and fermentation leading to CBP; a few research teams are hunting for new microbes with enhanced hydrolytic capacities; others are exploring the possibility of incorporating advance non-biological steps into the process (Lynd et al. 2008).

## Consolidated bioprocessing (CBP)

CBP employs microbes to perform all the four biologically-mediated transformations viz. the production of saccharolytic enzymes, the hydrolysis of carbohydrate components present in biomass to simple sugars, the fermentation of hexose sugars and the fermentation of pentose sugars in a single step (Fig. 1) (Lynd et al. 2005; Amore and Faraco 2012; Schuster and Chinn 2013). Thus, it offers the potential for lowering the cost and enhancing the efficiency of bioethanol production, as compared to independent hydrolysis and fermentation steps. Independent hydrolysis and fermentation can lead to end-product inhibition by sugars and acetates produced via hydrolysis (Wright et al. 1988). These inhibitors disrupt the fermentation capabilities of the ethanologen and result in substantial yield reductions. CBP might eliminate problems attributed with glucose accumulation and other such inhibitors (Olofsson et al. 2008; Dashtban et al. 2009). Another advantage of using CBP is that glucose does not need to be separated from the lignin fraction following the hydrolysis step, thus minimising sugar loss (Olofsson et al. 2008).

Increasing evidence suggests that CBP may be feasible (Ali et al. 2012b, 2013, 2014; Xu et al. 2009; Xiros and Christakopoulos 2009; Bokinsky et al. 2011; Hyeon et al. 2011; Okamoto et al. 2014). Ever since the concept of CBP was proposed in 1996, CBP research has focused on the development of new and even more effective CBP microorganisms, which has been a key challenge (Lynd et al. 2005). Bacteria and yeast have been the primary candidates for CBP research and some progress has been made in this regard (Lynd et al. 2005; Amore and Faraco 2012; den Haan et al. 2015). Fungi have not been widely proposed as CBP microorganisms, but there are a few recent reports of researchers developing strains of the fungi *Fusarium oxysporum* and *Trichoderma reesei* with enhanced CBP potential (Ali et al. 2012b, 2013, 2014; Xiros and Christakopoulos 2009; Xu et al. 2009; Huang et al. 2014).

There are several drawbacks associated with CBP. Microbial growth, enzymatic hydrolysis and the fermentation phases are carried out synchronically and it is very difficult to find culture conditions that are optimal for all these processes. Traditionally, proponents of CBP processes have identified two approaches capable of producing industrially-viable microbial agents for CBP. These are: (i) engineering a cellulase producer, such as *Clostridium thermocellum*, to be ethanologenic, and (ii) engineering an ethanologen, such as *S. cerevisiae* or *Zymomonas mobilis*, to be cellulolytic (Xu et al. 2009). Efforts have so far been focused mainly on the second approach. The bacteria *Z. mobilis* (Linger et al. 2010; Luo and Bao 2015), *Escherichia coli* (Ingram et al. 1999; Tao et al. 2001; Ko et al. 2013) and *Klebsiella oxytoca* (Tran et al. 2011; Wood et al. 2005), and the yeasts *S. Cerevisiae* (Hong et al. 2014; Xu et al. 2014; van Zyl et al. 2007), *Pachysolen tannophilus* (Slininger et al. 1987), *Pichia stipitis* and *Candida shehatae* (Prior et al. 1989) have been modified in order to improve their performance in CBP. But there are various difficulties and challenges in the conversion of a candidate microorganism using gene transfer technology. These include the adverse effects of the co-expression of multiple heterologous genes on cell performance, the modulation of simultaneous co-expression of multiple genes at the transcription level and improper folding of some secretory proteins (Xu et al. 2009). For instance several studies have found that the heterologous expression of cellobiohydrolases I and II in *S. cerevisiae* resulted in poor levels of conversion of crystalline cellulose degradation (den Haan et al. 2007; Hong et al. 2003; Chow et al. 1994). Thus, the search for a native CBP agent is very much essential and their study will also increase the arsenal of genes available for genetic manipulation of microbes currently being developed for CBP.

## The potential of filamentous fungi as CBP agents

Despite recent advances in engineering cellulases to be more efficient and less costly, the complete saccharification of lignocellulose still requires a very long time for digestion and high loadings of enzyme (30–50 mg enzyme g^−1^ of crystalline cellulose) (Xu et al. 2009). Thus, a biorefinery consuming thousands of tons of biomass per day will require many tons of cellulase enzymes to operate. Only fungi appear to be able to produce sufficient amounts of cellulase to meet this need. For example, *T. reesei* is reported to be able to produce more than 100 g cellulase enzyme per litre of culture broth (Cherry and Fidantsef 2003; Vitikainen et al. 2010). In comparison, the most productive cellulolytic bacteria produce only a few grams per litre (Xu et al. 2009). The ability to produce and secrete enzymes of complex structure, such as the cellobiohydrolase I, requires a robust secretion system, including the endoplasmic reticulum and the golgi complex within the cytosol of the cell (Xu et al. 2009). Bacteria do not normally have such systems and it may be difficult or impossible to engineer these organisms to produce cellulolytic enzymes in sufficient quantities for a biorefinery (Xu et al. 2009).

Microorganisms used in the CBP of lignocellulose to bioethanol must display high levels of alcohol/sugar tolerance, thermotolerance and tolerance to other inhibitors that may be produced as a result of either pretreatment or the CBP process itself. An ability to utilise multiple sugars is also a prerequisite. The proposed use of filamentous fungi in the CBP process goes a long way towards satisfying these requirements. *T. reesei* is considered to be a one of the best CBP fungi due to its capacity to produce and secrete lignocellulolytic enzymes. The extensive knowledge of its physiology and cellulolytic machinery (Kubicek et al. 2009; Schmoll et al. 2010; Silva-Rocha et al. 2014) and the availability of a range of tools for its genetic manipulation enhance its preference as a CBP organism (Kück and Hoff 2010). Yet, *T. reesei* also presents some challenges that must be addressed before it can become an efficient CBP organism. Its main limitations are the low ethanol yield and rate of production, low ethanol tolerance, and difficulties during fermentation associated with its cell morphology (Xu et al. 2009). Another restriction is the fact that genes encoding enzymes crucial for glycolysis are repressed in the absence of oxygen (Bonaccorsi et al. 2006), which limits its growth without oxygen. Xu et al. (2009) demonstrated that *T. reesei* can survive, but not thrive, and convert soluble sugars to ethanol under anaerobic or microaerobic conditions. In addition to *T. reesei*, there are alternative fungi presenting a significant potential to become a CBP organism. These include *Fusarium oxysporum* (Ali et al. 2012b; Christakopoulos et al. 1989)*, Mucor indicus* (Karimi et al. 2006), *Monilia sp.* (Gong et al. 1981), *Rhizopus oryzae* (Karimi et al. 2006; Battaglia et al. 2011), *Paecilomyces* sp. (Wu 1989), *Aspergillus oryzae* (Skory et al. 1997), *Neocallimastix patriciarum* (Wang et al. 2011) and *Neurospora crassa* (Dogaris et al. 2009). These can all produce bioethanol from cellulosic material. Most of these filamentous fungi possess the ability to assimilate and metabolise numerous sugars, both hexose and pentose types (Taherzadeh and Karimi 2007). Furthermore, these fungi have a greater degree of thermotolerance than many bacteria and can grow at 37 °C, which is closer to the optimal temperature of enzymatic hydrolysis (40–50 °C) (Millati et al. 2005). This advantage was considered to improve theoretical ethanol yields by as much as 15 % compared to *S. cerevisae* (enzymatic hydrolysis followed by fermentation) when using *M. indicus* and *R. oryzae* in CBP of dilute-acid pretreated rice straw (Karimi et al. 2006). Bioethanol production from cellulosic biomass by various filamentous fungi has been summarised in Table 1.

**Table 1:** The potential of filamentous fungi as CBP agents

Organism	Number of strains tested	Theoretical ethanol yield from biomass (%)^a^				Reference
		Cellulose	Other lignocellulose	Glucose	Xylose	
*Aspergillus awamori*	2	1.5–1.1	–	28.2–23.5	5.4–3.5	Skory et al. (1997)
*A. foetidus*	1	1.1	–	20.3	13.3	Skory et al. (1997)
*A. niger*	1	0.7	–	22.3	4.7	Skory et al. (1997)
*A. oryzae*	4	3.1–2.3	–	95.6–62.3	18.4–10.1	Skory et al. (1997)
*A. sojae*	6	1.5–2.7	–	56.4–31.3	21.1–8.2	Skory et al. (1997)
*A. tamari*	5	2.3–0.3	–	72.9–38.4	13.7–11.3	Skory et al. (1997)
*Fusarium oxysporum*	3	89.2 (alkali-treated)	–	80	48	Christakopoulos et al. (1989)
*F. oxysporum*	1	–	83 (ball milled wheat straw); 67 (alkali treated wheat straw)	–	–	Christakopoulos et al. (1991a, b)
*F. oxysporum*	17		19.7 (untreated wheat straw); 80.2 (alkali treated wheat straw)			Ali et al. (2012b)
*Flammulina velutipes*	1	–	57.2 (sweet sorghums)	–	–	Mizuno et al. (2009)
*Fomitopsis palustris*	1	88.2 (cellobiose)	–	90.2	–	Okamoto et al. (2011b)
*Gloeophyllum trabeum*	1		10.4 (pretreated corn fiber)	–	–	Rasmussen et al. (2010)
*Mucor indicus*	1	61 (avicel)	68 (rice straw)	–	–	Karimi et al. (2006)
*Monilia* sp.	1	60	–	–	–	Gong et al. (1981)
*Mucor hiemalis*	1	–	80 (pretreated sweet sorghum bagasse)	–	–	Goshadrou et al. (2011)
*Neurospora crassa*	1	60	–	–	–	Rao et al. (1983)
*N. crassa*	1	100 (avicel) 91 (alkali-treated)	54 (alkali treated sugarcane bagasse)	96.9	64.2	Deshpande et al. (1986)
*N. crassa*	1	–	11.76 (sorghum bagasse)	–	–	Dogaris et al. (2009)
*Phanerochaete chrysosporium*	1	–	6.8 (corn fiber)	–	–	Shrestha et al. (2010)
*Phlebia* sp.	1		65.7 (alkali treated sugarcane bagasse)			Khuong et al. (2014)
*Paecilomyces* sp.	1		18 (wheat bran and brewers spent grain mix)	61.8	82	Zerva et al. (2014)
*Paecilomyces* sp.	1	78.4	–	–	78	Wu (1989)
*Rhizopus javanicus*	3	5–1.9	–	92.1–48.6	45.8–6.6	Skory et al. (1997)
*R. oryzae*	6	5.4–.7	–	99.5–58.4	42.3–3.9	Skory et al. (1997)
*R. oryzae*	1	76 (avicel)	74 (rice straw)	–	–	Karimi et al. (2006)
*Trichoderma harzianum*	1	7.8	–	19.6	2.3	Stevenson and Weimer (2002)
*T. reesei*	1	–	14.5 (corn fiber)	–	–	Shrestha et al. (2010)
*Trametes hirsuta*	1	–	78.8 (wheat bran), 57.4 (rice straw)	96	86.2	Okamoto et al. (2011a)

^a^Maximum theoretical yield was calculated based on the fact that 0.51 g ethanol and 0.49 g of CO_2_ are yielded from 1 g of glucose. The % theoretical yield was calculated based on the sugar content of lignocellulosic material. When sugar content was not mentioned in the original reference, it is calculated based on average composition of lignocellulose which can theoretically produce 0.336 g ethanol g^−1^ of biomass (Szczodrak 1988)

## *Fusarium oxysporum* as a potent CBP agent

*Fusarium oxysporum* is a filamentous soil-borne fungus that is more widely regarded as a phytopathogen responsible for vascular wilt disease in a variety of different plant species or as a mycotoxin-producing contaminant of human and animal food (Di Pietro et al. 2003). The majority of crops cultivated worldwide are hosts to a pathogenic form of *F. oxysporum*. It is among the handful of microbial species that are known to have the enzymatic systems needed to break down cellulose and hemicellulose and to ferment the released hexoses and pentose sugars to bioethanol in a single step (Ali et al. 2012b; Christakopoulos et al. 1989; Panagiotou et al. 2005b; Singh et al. 1992; Suihko 1983). These and other associated characteristics render it relatively efficient for the CBP of lignocellulose to bioethanol. This ability stems from the relatively high levels of cellulases and xylanases produced by *F. oxysporum* (Christakopoulos et al. 1989, 1996a, b; Panagiotou et al. 2003). *Fusarium oxysporum* also exhibits high levels of tolerance to sugars, ethanol and inhibitors such as acetate (Hennessy et al. 2013; Singh and Kumar 1991). *Fusarium oxysporum*-mediated fermentation of glucose is unaffected until ethanol concentrations reach 4.5–5.0 % in the reactor (Enari and Suihko 1983). Acetate is a major inhibitory compound produced by microbes during the ethanol fermentation process, but growing cells of *F. oxysporum* are capable of reducing acetate to ethanol (Enari and Suihko 1983).

*Fusarium oxysporum* requires an aerobic growth phase (for initial fungal growth) followed by an oxygen-limited fermentation phase in order to produce ethanol from glucose (Panagiotou et al. 2005a). To optimise the performance of *F. oxysporum* during the fermentation phase, a limited oxygen supply of 18–20 % is required (Panagiotou et al. 2005d). Such level of oxygen will facilitate growth and the breakdown of acetate by growing cells (Enari and Suihko 1983). As shown in Table 1, the highest reported bioethanol yield from any unprocessed lignocellulosic material by a filamentous fungus to date was achieved by *F. oxysporum* strain 11C, producing up to 80 mg bioethanol g^−1^ wheat straw/bran (Ali et al. 2012b). With alkali-treated straw as the substrate, the yield increased to 326 mg bioethanol g^−1^ wheat straw/bran, a theoretical yield of 80.2 %. Although still below industrial exploitable yields, this yield is higher than that reported for other fungi grown on pre-treated agricultural waste (Deshpande et al. 1986; Mizuno et al. 2009; Karimi et al. 2006; Okamoto et al. 2011a, b; Goshadrou et al. 2011). Interestingly, the ability of *F. oxysporum* to produce ethanol during CBP was found not be reflective of either cellulose or alcohol dehydrogenase (ADH) activity (Ali et al. 2012b). Explorative work by Ali et al. (2014) identified a large consortium of *F. oxysporum* genes (<210) that were upregulated in *F. oxysporum* strain 11C and may have conferred this strain with an enhanced capacity for bioethanol production from lignocellulosic residues. These genes were identified by comparing the transcriptome of *F. oxysporum* strain 11C with that of a low efficacy *F. oxysporum* strain. Many of the genes identified and their encoded proteins could be assigned to various categories such as carbohydrate metabolism, energy, protein and sugar transport and detoxification. Furthermore, a consortium of novel genes were discovered that had no direct link to saccharification or fermentation but were shown to be activated during CBP, highlighting the complexity of the process (Ali et al. 2014). Another promising strain, *F. oxysporum* strain F3, has been reported to directly convert alkali-treated and ball-milled wheat straw to bioethanol, producing 67.8 and 83 % of theoretical yield, respectively (Christakopoulos et al. 1991a, b). The same strain has been reported to yield 109 g bioethanol kg^−1^ of dry alkali- pretreated brewer’s spent grain under microaerobic conditions, corresponding to 60 % of the theoretical yield (Xiros and Christakopoulos 2009). Further studies were done with this particular *F. oxysporum* strain to saccharify and co-ferment wheat straw with *S. cerevisiae* (Panagiotou et al. 2011); yields of bioethanol were very promising (40 g kg^−1^ of pretreated wheat straw).

## The CBP metabolic pathway of *F. oxysporum*

**Fig. 2:**
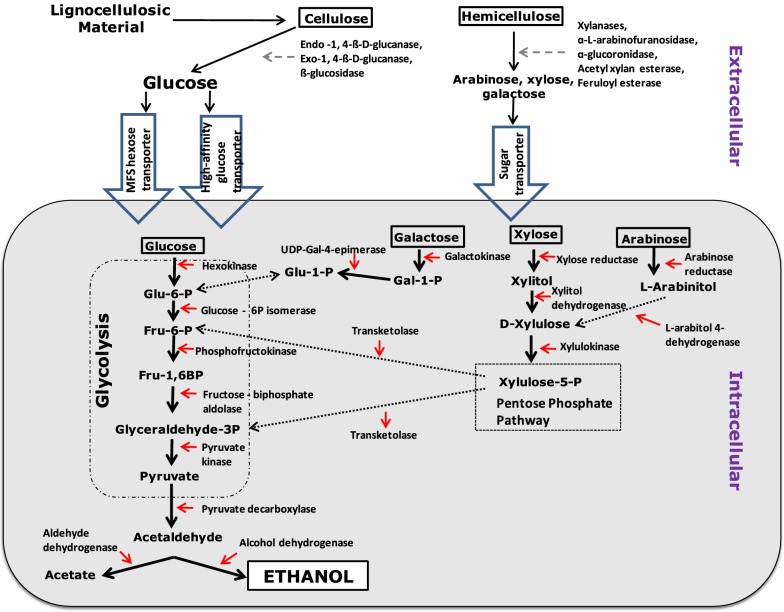
The metabolic pathway involved in the bioconversion of lignocellulosic material to bioethanol by *Fusarium oxysporum*. *Glu-6-P* Glucose-6-Phosphate, *Fru-6-P* Fructose-6-Phosphate, *Fru-1, 6BP* fructose-1, 6-biphosphate, *glyceraldehyde-3P* glyceraldehyde-3-phosphate, *Glu-1-P* glucose-1-phosphate, *Gal-1-P* galactose-1-phosphate, *Xylulose-5-P* xylulose-5-phosphate

During CBP the first metabolic step for any microbial agent is the production of cellulases and hemicellulases followed by extracellular secretion of these enzymes for the purpose of substrate hydrolysis. Celluloytic and hemicelluloytic enzymes produced by *F. oxysporum* are used to breakdown cellulose and hemicellulose to their simple sugar derivatives which can then be imported into the cell by sugar transporters. Glucose enters glycolysis on immediate arrival inside the cell. Conversely, the hemicellulosic sugars (xylose, galactose, arabinose) must enter alternative metabolic pathways where they are enzymatically converted to glycolytic intermediates, allowing them to enter glycolysis and undergo subsequent fermentation to bioethanol (Fig. 2). Cellulase is a multienzyme complex consisting of endo-l,4-β-d-glucanase, exo-l,4-β-d-glucanase and β-glucosidase. Synergistic activity of these enzymes is required for the complete hydrolysis of insoluble cellulose (Halliwell 1961). *Fusarium oxysporum* has been shown to produce all three of these enzymes (Ali et al. 2012b; Christakopoulos et al. 1989; Kumar et al. 1991). Cellulase production by *F. oxysporum* is a function of the level of the soluble hydrolysis products (glucose and cellobiose) that can be utilised by the fungal cells; this in turn is dependent on the rate of adsorption of endoglucanase onto the cellulose and rate of hydrolysis of this substrate (Targonski and Szajer 1979; Singh et al. 1991).

Three endoglucanases and one β-glucosidase from *F. oxysporum* strain F3 has been purified and fully characterised (Christakopoulos et al. 1994, 1995a, b, c). The significantly higher β-glucosidase activity of this particular strain prevented the inhibitory effect of cellobiose on cellulase activity (Panagiotou et al. 2005b) and a low molecular mass endoglucanase may have played an important role in cellulose degradation as it can penetrate through the cellulose fibre easily (Christakopoulos et al. 1995c). Interestingly, in the case of *F. oxysporum* strain 11C, the capacity to produce ethanol during fungal CBP was not linked to cellulase activity (Ali et al. 2012b). Alternatively, a large consortium of up-regulated genes encoding proteins assigned to various categories, including carbohydrate metabolism, energy, protein and sugar transport and detoxification were identified as likely factors in *F. oxysporum*-mediated CBP (Ali et al. 2014).

The variable structure and organisation of hemicellulose necessitates the concerted action of many enzymes for its complete degradation. The catalytic modules of hemicellulases are either glycoside hydrolases (GHs) that hydrolyse glycosidic bonds, or carbohydrate esterases (CEs), which hydrolyse ester linkages of acetate or ferulic acid side groups (Shallom and Shoham 2003). Shallom and Shoham (2003) described thirteen different enzymes required for the complete hydrolysis of hemicellulose; various hemicullases from *F. oxysporum* has been extensively studied and this strain can produce most of the enzymes required for the complete hydrolysis of hemicellulose (Singh et al. 1995; Christakopoulos et al. 1996a; Cheilas et al. 2000; Panagiotou et al. 2003; Anasontzis et al. 2011; Ali et al. 2012b, 2013).

Sugar transport across the cell membrane is the first step in the metabolism of sugars and this occurs through facilitated diffusion (Jeffries 1983; Kim et al. 2003). In yeast there are twenty different glucose transporter genes and expression of these transporters are regulated by the glucose concentration (Ali et al. 2013; Ozcan and Johnston 1995). Brandao and Loureiro-Dias (1990) observed that sugar transport of *F. oxysporum* was under the same regulatory mechanism as that of yeast and other eukaryotic microorganisms. It has been observed that the rate of sugar transport determines the rate of anaerobic fermentation and ethanol production/tolerance in yeast (Leão and Uden 1985; Alexandre et al. 2001) and *Fusarium* (Ali et al. 2013; Hennessy et al. 2013). Indeed, manipulation of a *Hxt* gene encoding for a high affinity glucose transporter in *F. oxysporum* was found to have a profound effect on the overall activity and productivity of the fungus during CBP, with *Hxt* overexpression mutants achieving the highest yield to date of a fungus from pre-treated agricultural waste (Ali et al. 2013). Transport of d-xylose has been found to be related to d-glucose transport, since the influx of d-xylose or d-arabinose is more rapid in the presence of glucose under anaerobic conditions than under aerobic conditions (Jeffries 1983). Concomitantly, it was also observed that overexpression of the *Hxt* gene in *F. oxysporum* increased not only glucose uptake rates but likewise xylose uptake rates resulting in enhanced fermentation rates (Ali et al. 2013). In regards to tolerance, transcriptional studies have revealed the functional role filamentous fungal sugar transporters play in times of alcohol stress (Alexandre et al. 2001; Chandler et al. 2004; Reyes et al. 2011; Hennessy et al. 2013).

In yeast and fungi, glucose is metabolised to ethanol via the Embden-Meyerhof-Parnas (EMP) pathway (Rose and Harrison 1970). Theoretically, 0.51 g ethanol and 0.49 g of CO_2_ are yielded from 1 g of glucose. However, the real ethanol and CO_2_ yields are 0.46 g and 0.44 g respectively, since 0.10 g of glucose is metabolised for biomass production (Singh and Kumar 1991). In the case of *F. oxysporum,* the highest bioethanol yield to date from glucose was recorded as 0.409 gg^−1^ (Panagiotou et al. 2005d). Panagiotou et al. (2005b) has analysed the intracellular metabolic profile of *F. oxysporum* strain F3 under aerobic and anaerobic conditions. He observed that there was a high glycolytic flux under anaerobic growth conditions, characterised by a high efflux of glyceraldehyde-3-phosphate (G3P) and fructose-6-phosphate from the pentose phosphate pathway (PPP) to the EMP pathway, resulting in the highest bioethanol production under these conditions. The amino acid profile of fermenting *F. oxysporum* cells clearly suggested that the TCA cycle was primarily active under aerobic cultivation. On the other hand, the presence of high levels of γ-amino-*n*-butyric acid (GABA) under anaerobic conditions suggested a functional GABA bypass and a possible block in the TCA cycle under these conditions (Panagiotou et al. 2005d). An accumulation of sedoheptulose-7-P was observed indicating a barrier in the PPP that affects the production of NADPH and results in the production of acetate. Furthermore, the presence of high levels of GABA under anaerobic conditions suggest a functional GABA bypass and a possible block in the TCA cycle, which may also contribute to acetate production (Panagiotou et al. 2005d). It was interesting to observe that *F. oxysporum* does not co-metabolise xylose with glucose during anaerobic growth and starts only after glucose exhaustion (Panagiotou et al. 2005c). Under anaerobic conditions xylitol is the main product of xylose metabolism (Panagiotou et al. 2005c). Therefore, it can be predicted that a metabolic engineering approach to block the acetate and xylitol pathway will dramatically boost bioethanol yielded via CBP by *F. oxysporum*.

## A roadmap for enhanced CBP activity

*Fusarium oxysporum* is known for its slow rate of growth which is a major drawback for use as a CBP ethanologenic agent as the microorganism takes longer to reach the required critical mass before the transition to anaerobic conditions for fermentation. Quickening the maximum specific growth rate of *F. oxysporum* has been successfully achieved recently through the overexpression of both phosphoglucomutase and transaldolase (Anasontzis and Christakopoulos 2014). Further improvements to speed up the growth rate of *F. oxysporum* would be a major breakthrough in the quest towards designing the ultimate CBP microbial agent. As *F. oxysporum* and other CBP agents are already adept at fermenting the cellulosic fraction of plant biomass, the greatest gains in bioethanol yield will likely come from increased catabolism of the under-utilised hemicellulosic fraction. Therefore, the tactical co-expression of hemicelluloytic genes in a designer strain of *F. oxysporum* such as the gene encoding for a α-l-arabinofuranosidase (*AbfB*) (Ali et al. 2014) involved in degradation of hemicellulose to its simple sugar derivatives with the previously highlighted *Hxt* gene (involved in sugar transport) (Ali et al. 2013) could drastically improve hemicellulosic fermentation rates. Development of a designer strain with the ability to metabolise all hemicellulosic sugars would represent a significant breakthrough for the lignocellulosic bioethanol industry.

During CBP, *F. oxysporum* produces large amounts of acetic acid, an unwanted by-product of the fermentation process that impedes the bioethanol productivity of the fungus. Higher transcript levels of a gene encoding acetyl-CoA hydrolase has been observed in *F. oxysporum* strain 11C before which is likely associated with the increased acetic acid production of the strain (Ali et al. 2012b, 2014). The targeted disruption of this gene and other genes that are involved in by-product formation could serve to further enhance *F. oxysporum*’s CBP efficacy to minimize the production of undesirable by-products such as acetic acid during lignocelluloytic bioconversion.

*Fusarium oxysporum* is a species with high morphological and physiological variation and its ubiquitous presence in ecological activities worldwide indicate a highly diverse and important role in nature. Though the fungus is highly successful as a saprophyte in soil, as stated earlier, most of the interest in it arises from its ability to cause detrimental diseases on various economically important crops. Both plant pathogenic and non-pathogenic isolates could provide a rich source of industrial enzyme sources for bioethanol production (King et al. 2011). Unlike the pathogenic strains, very little is known about the genetic and molecular variation among the non-pathogenic *F. oxysporum* strains and these could potentially serve as a rich source of plant-degrading enzymes. Ali et al. (2012b, 2013) observed significant inter-strain divergence in regards to the capacity of different *F. oxysporum* strains to produce alcohol from wheat straw and identified 210 transcripts encoding proteins assigned to various categories, including carbohydrate metabolism, energy, protein and sugar transport and detoxification that were overexpressed in a high as compared to low efficacy *F. oxysporum* strains during CBP. The level of diversity recorded in the bioethanol production capacity among the isolates means that a targeted screening of populations of selected isolates could greatly improve bioprocessing yields, in terms of providing both new host strains and candidate genes for the bioethanol industry.

## Conclusion

Under the present predicted scenario of a food and fuel crisis, coupled with global warming, cellulosic bioethanol shows promise as an alternative to petroleum. Filamentous fungi such as *F. oxysporum* could be commercially competitive CBP agents, or components of their enzymatic arsenal could contribute to the development of a ‘designer’ CBP agent. CBP needs much robust microbial agents in order to bring this process to the level of industrial expectations. The most efficient CBP of lignocellulose will most likely be achieved using a consortium of enzymes contributed by several microbes that are either working in unison or as donors of genes pyramided into one or more ‘designer’ organisms. Approach for such a designer agent should include (i) increasing the ethanol yield, (ii) eliminating by-products, (iii) improving the tolerance to ethanol, and (iv) introduction of new metabolic pathways for assimilating lignocellulose sugars. Understanding all these components and the complexity of the networks involved will be important in selecting such a consortia or designing such an organism. A functional genomic or proteomic approach could help unfold the networks involved and would open up many more avenues for the improvement of CBP agents. Then synthetic biology can provide new tools to rewire the cell components (promoters, regulators, terminators, enzymes, operons, transporters, etc.) in order to reach the desired features for the production of economically viable biofuels. Although some successful examples were already reported in bacteria and yeast, a crucial remaining challenge is to apply these approaches in fungi, which have a tremendous potential since they are the effective producers of critical cellulases.
